# The Effect of Silicone Modification on the Thermo-Oxidative Degradation and Mechanical Property Retention of CFRP Laminates

**DOI:** 10.3390/polym18192405

**Published:** 2026-10-02

**Authors:** Junkang Xia, Yundong Ji, Jingang Liu, Dongfeng Cao, Wei Cai, Hongda Chen, Lei Wang, Shichao Wu, Shuxin Li

**Affiliations:** 1 State Key Laboratory of Advanced Technology for Materials Synthesis and Processing, Wuhan University of Technology, Wuhan 430070, China; 2 Hubei Key Laboratory of Theory and Application of Advanced Materials Mechanics, Wuhan University of Technology, Wuhan 430070, China; 3 Aviation Operations Service College, Air Force Aviation University, Changchun 130022, China; 4 Institute for Frontier and Interdisciplinary Innovation, Wuhan University of Technology, Wuhan 430119, China; 5 Wanhua Chemical Group Co., Ltd., Yantai 265505, China

**Keywords:** CFRP composites, silicone-modified epoxy, thermo-oxidative degradation, mechanical degradation, damage tolerance

## Abstract

The mechanical properties of CFRP laminates degrade significantly in extreme thermal environments above the onset temperature of thermo-oxidative degradation of the matrix. This study investigates the thermo-oxidative damage evolution and mechanical degradation mechanisms of CFRP laminates with silicone-modified epoxy (SME) and neat epoxy (EP) through thermogravimetric analysis, multi-scenario thermo-oxidative degradation tests, tensile/compressive measurements and multi-scale SEM observations. The results show that silicone modification reduces the peak mass loss rate from approximately 10 to 6 %·min^−1^; at 350 °C for 3 h, the mass loss of SME-based CFRP is about 42.3% lower than that of EP-based CFRP. Strengths under all loading configurations decay monotonically with degradation severity, with the strength of SME-based laminates exceeding that of the EP counterparts by up to 139.7% under the most severe condition. The C–Si–O hybrid network suppresses matrix degradation and fiber–matrix debonding, delaying crack initiation and propagation, thereby markedly enhancing the thermo-oxidative damage tolerance of the composites. This work provides data support for damage-tolerance design and safety assessment of composite structures in extreme thermal environments.

## 1. Introduction

With the rapid development of aerospace, including advanced energy and defense equipment technologies, carbon fiber-reinforced polymer (CFRP) composites have been extensively employed in extreme service environments bearing complex thermo-mechanical coupled loads, owing to their excellent specific strength, design flexibility and multifunctionality [1]. However, when the service temperature exceeds the onset temperature of thermo-oxidative degradation of the resin matrix, CFRP laminates undergo a complex thermal–chemo-mechanical response process involving multi-scale, multi-physics coupling [2,3]. Thermo-oxidative degradation and molecular chain scission of the epoxy matrix induce a series of irreversible micro-damage, including residual char formation, volatile gas release, initiation and propagation of micropores and microcracks, and progressive fiber–matrix interfacial debonding [4,5,6]. The accumulation of micro-damage not only leads to pronounced degradation of macroscopic mechanical properties, but also fundamentally alters the failure modes and load-bearing mechanisms of the material [7,8]. Therefore, revealing the damage evolution of CFRP during high-temperature thermo-oxidative degradation and its intrinsic correlation with mechanical property degradation has become an urgent requirement for ensuring the structural safety of hot-end components and achieving damage tolerance design of composite structures [9,10].

In recent years, the property degradation mechanisms of resin matrix composites in high-temperature environments have been extensively investigated. It has been demonstrated that high-temperature exposure causes softening and degradation of the resin matrix, significantly weakening the fiber–matrix interfacial bonding strength and reducing the load transfer efficiency [11,12]; meanwhile, the synergistic effect of thermal stress and thermo-oxidative shrinkage induces the nucleation and propagation of matrix microcracks, which gradually evolve into severe damages such as interfacial debonding and interlaminar delamination with prolonged thermo-oxidation time [13,14]. The aforementioned micro-deterioration directly modifies the failure characteristics of composites under complex loads including tension, compression and shear [15,16]. Furthermore, the mechanical properties of FRP composite structures are not only influenced by matrix modification, but are also closely related to the structural configuration of the reinforcement and the face sheets [17]. Although progress has been made in degradation kinetic modeling and residual strength prediction [18], most studies remain limited to qualitative descriptions of transient high-temperature softening mechanisms or property characterization at a single temperature point. Systematic quantitative correlations between damage evolution and mechanical property degradation during long-term thermo-oxidation are still lacking; in particular, the intrinsic mechanism by which multi-scale thermo-oxidative damages (voids, debonding, delamination, etc.) govern the loss of macroscopic load-bearing capacity and the transition of failure modes remains unclear.

To improve the high-temperature resistance of epoxy resins, flame-retardant modification technologies have advanced considerably in recent years [19]. Compared with conventional halogenated and phosphorus-based flame retardants, silicone modification has attracted considerable attention due to its unique char-forming mechanism and environmental friendliness. During thermo-oxidative degradation, silicon in silicone-modified epoxy can migrate to the material surface and form a dense silicon-rich char layer (siloxane protective layer), which inhibits the further development of degradation reactions toward the material interior by virtue of its excellent thermal insulation and oxygen isolation barrier effects [20,21,22]. For instance, Hamdani et al. [23] systematically demonstrated the flame-retardant mechanism of silicon-based materials and the shielding effect of the silicon-rich char layer; Heng et al. [24,25] confirmed through the synthesis of silicon-containing epoxy resins that the incorporation of silicon can significantly enhance the thermal stability and char yield of the matrix. However, existing studies have mainly focused on the improvement in intrinsic flame retardancy and heat resistance of the resin matrix by silicone modification, whereas the residual mechanical property retention capability of silicone-modified composites after severe high-temperature exposure remains largely unexplored [26,27]. Therefore, systematic work [17] is urgently needed to quantitatively characterize the evolution of strength, modulus and failure modes of modified CFRP under varying degrees of thermo-oxidative degradation.

In view of this, the present study takes CFRP laminates with hydroxyl-terminated phenylpropyl polysiloxane (PPPS)-modified epoxy (hereinafter referred to as silicone-modified epoxy, SME) as the research object, with the unmodified epoxy (EP) system as a reference, and systematically investigates their damage evolution and mechanical property degradation mechanisms under high-temperature thermo-oxidative degradation. First, thermogravimetric analysis (TGA) is employed to comparatively characterize the differences in thermo-oxidative degradation kinetic behavior between the two resin systems; second, thermo-oxidative degradation scenarios encompassing various degrees of degradation severity are designed, and macroscopic tensile and compressive tests are conducted on the post-degradation laminates, combined with multi-scale experimental characterization to reveal how thermo-oxidative damage governs the load-bearing capacity attenuation and failure mode transition of CFRP. This study aims to provide data support for damage tolerance design, life prediction and safety assessment of silicone-modified composites in extreme thermal environments.

## 2. Experimental Design, Fabrication, Testing and Characterization

### 2.1. Resin Modification Strategy and Thermogravimetric Analysis

Epoxy resin (EP; Sinopec Baling Petrochemical Co., Ltd., Yueyang, China) and hydroxyl-terminated phenylpropyl polysiloxane (PPPS; Dow Corning (Shanghai) Co., Ltd., Shanghai, China) were weighed at a mass ratio of 80:20 (EP/PPPS) and dissolved in acetone under continuous stirring at room temperature until complete dissolution was achieved. A 250 mL three-necked flask, equipped with a reflux condenser, a mechanical stirrer and a precision thermometer, was placed in a constant-temperature water bath preheated to 25 °C. High-purity nitrogen was continuously purged through the reflux condenser throughout the reaction to eliminate oxygen interference. The prepared PPPS solution and the epoxy resin were charged into the flask and stirred vigorously until a homogeneous dispersion was obtained. Subsequently, the catalyst dibutyltin dilaurate (DBTDL; Sinopharm Chemical Reagent Co., Ltd., Shanghai, China) was added, and the reaction was carried out at 25 °C and 200 rpm for 2 h; phosphoric acid was then introduced, and the reaction temperature was raised to 40 °C for an additional 1 h.

**Figure 1:**
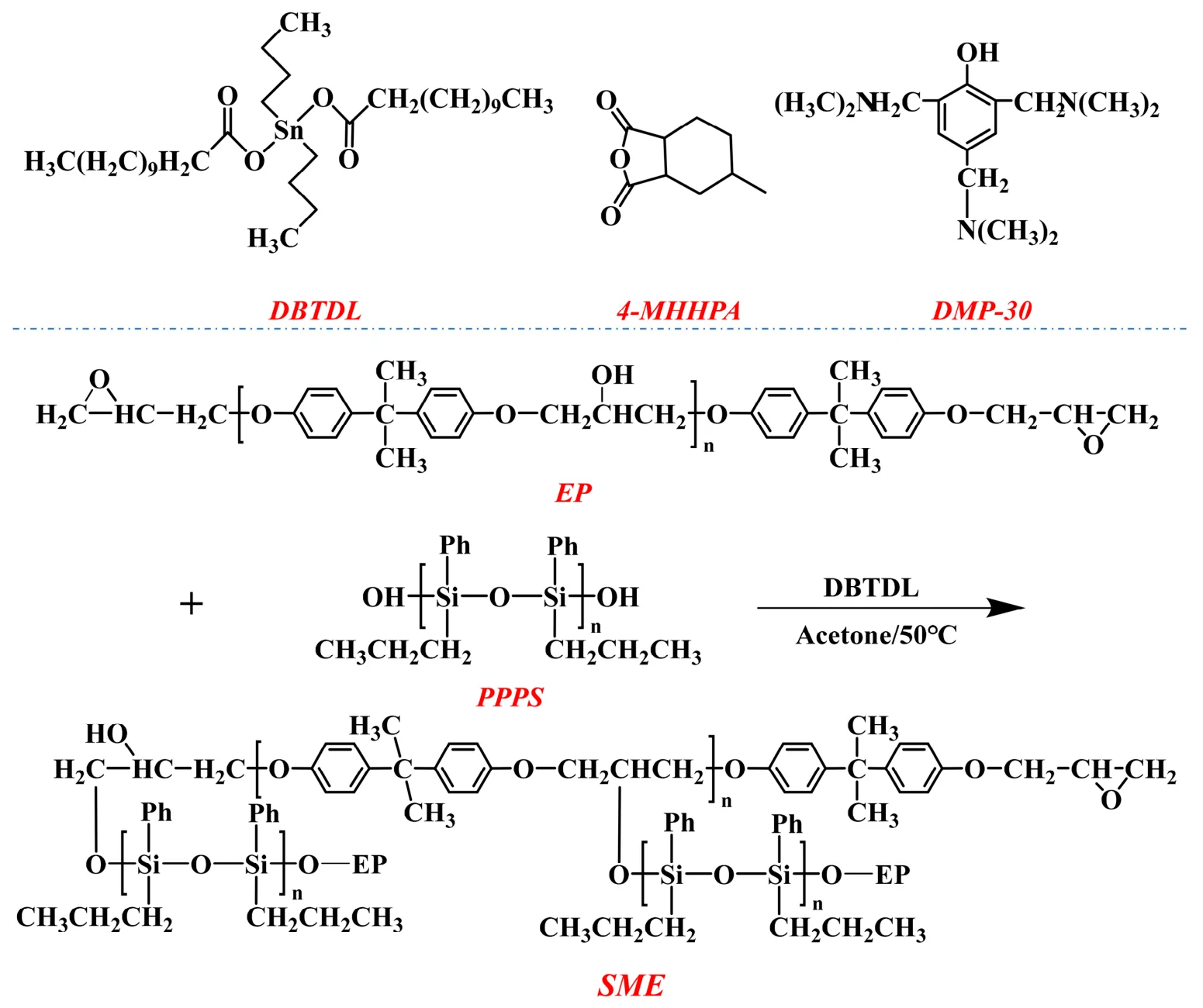
Synthesis of PPPS-modified epoxy resins.

After the reaction, the mixture was immediately transferred into a disposable container and the initial mass was recorded without delay. The sample was then placed in a vacuum oven and dried at 100 °C under -0.1 MPa (gauge pressure) with the vacuum pump running continuously to remove the solvent. The sample was removed and weighed every 2 h after cooling to room temperature, and the drying process was terminated when the mass difference between two consecutive weighings was less than 0.1%, which was regarded as reaching constant weight, indicating the complete removal of volatile components such as acetone. The molecular structure of the resulting product is shown in Figure 1. The nomenclature, composition and the corresponding proportions of curing agent and accelerator for the modified resins [21,28] are listed in Table 1. Among them, 4-MHHPA (Shanghai Aladdin Biochemical Technology Co., Ltd., Shanghai, China) was used as the curing agent and DMP-30 (Sinopharm Chemical Reagent Co., Ltd., Shanghai, China) as the accelerator.

To systematically investigate the differences in high-temperature thermal stability between the two resin systems, thermogravimetric analysis (TGA) was conducted on the neat epoxy resin (EP) and the silicone-modified epoxy resin (SME). The tests were performed on a NETZSCH STA 449 F5/F3 Jupiter simultaneous thermal analyzer (NETZSCH-Gerätebau GmbH, Selb, Germany) under an air atmosphere, with a heating rate of 10 °C/min over a temperature range from room temperature to 1000 °C. This part of the paper aims to provide a basis for determining the exposure temperatures in the subsequent experiments. 

### 2.2. Fabrication of Silicone-Modified CFRP Composites

**Figure 2:**
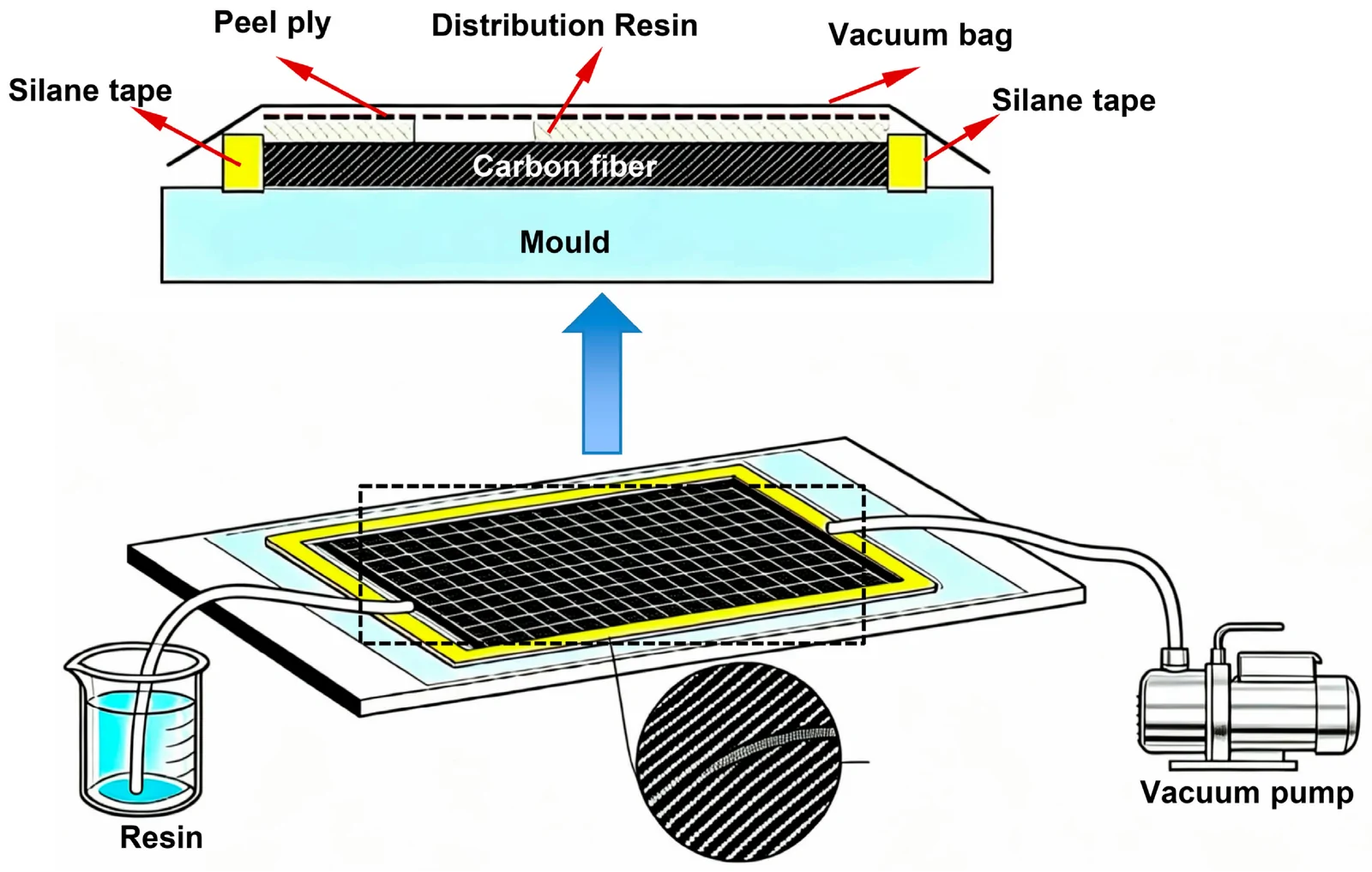
Schematic of vacuum infusion molding for CFRP composites.

Using the neat epoxy (EP) and silicone-modified epoxy (SME) as matrices and T700 unidirectional carbon fiber fabric as the reinforcement, CFRP laminates were fabricated via the vacuum infusion process [29], as schematically illustrated in Figure 2. Four basic mechanical tests—0° tension, 0° compression, 90° tension and 90° compression—were performed on the specimens after high-temperature thermo-oxidative treatment.

The fabrication procedure comprised the following key steps. First, hand lay-up was carried out with carbon fiber plies oriented at either 0° or 90° and stacked layer by layer; specifically, the laminates for 0° tension and 0° compression consisted of 5 and 24 plies, respectively, while those for 90° tension and 90° compression consisted of 10 and 24 plies, respectively, with the thickness of each panel conforming to the corresponding ASTM standards [30,31]. After lay-up, the preform was vacuum-bagged, leak-checked, and infused with resin under vacuum, ensuring thorough impregnation of the fibers. Both laminate types were fabricated using identical processing parameters to ensure comparability. The resin infusion was carried out at room temperature under a vacuum of −0.1 MPa (gauge pressure), and the laminates were cured in a convection oven following the schedule of 100 °C/2 h + 150 °C/5 h, with compaction provided solely by the vacuum bag. Only the resin matrix differed between the two laminate systems (neat EP vs. SME), while the fiber type (T700 carbon fiber), the lay-up sequence, and the target fiber content were kept identical. Following curing and demolding, the CFRP panels for post-degradation 0° tension, 0° compression, 90° tension and 90° compression tests had thicknesses of (1.0 ± 0.1) mm, (4.8 ± 0.1) mm, (2.0 ± 0.1) mm and (4.8 ± 0.1) mm, respectively.

The void content of the as-fabricated laminates was measured to verify the consistency of the two laminate systems. Five specimens were randomly cut from each panel, and the void content was determined by using the Archimedes method according to ASTM D2734 [32]. The measured void contents of the EP and SME laminates were 1.22% and 1.41%, respectively, both falling within the typical low-porosity range of the vacuum infusion process (≤2%), with no statistically significant difference between the two (*p* > 0.05). Combined with the identical fiber type, lay-up sequence and processing parameters described above, this confirms that the two laminate systems are of comparable quality, and the differences in mechanical properties reported below can therefore be attributed primarily to the resin matrix rather than to differences in laminate quality.

### 2.3. Thermo-Oxidative Degradation Protocols and Microstructural Characterization

It should be emphasized that the exposure regimes were designed to represent distinct, quasi-stabilized degrees of thermo-oxidative degradation, rather than equivalent exposure durations. To verify the approach to kinetic saturation, supplementary specimens were exposed at 250 °C for up to 168 h with weighing at 24 h intervals. At 250 °C, preliminary weighing showed that the mass loss approached a plateau toward the end of the 72 h exposure, indicating kinetic saturation consistent with diffusion-limited oxidation; the duration of 3 days was therefore selected as a quasi-saturated low-severity state. At 300 °C and 350 °C, degradation kinetics were markedly faster, and a holding time of 3 h was sufficient to reach clearly distinguishable and more advanced degradation states (mass losses of approximately 5–7% and 10–18%, respectively). Accordingly, the six scenarios in Table 2 are organized by degradation severity (characterized by cumulative mass loss), with temperature and exposure time serving as the means to realize each degradation state.

Based on the thermogravimetric analysis (TGA) results, the initial degradation temperature of 200 °C in air was adopted as the boundary of degradation kinetic behavior, and three degradation scenarios were established accordingly: 250 °C, 300 °C and 350 °C. Among them, 250 °C/3 d represents a mild, quasi-saturated degradation state; 300 °C/3 h and 350 °C/3 h represent moderate and severe degradation states, respectively. CFRP panels that passed ultrasonic C-scan nondestructive inspection were placed horizontally in the constant-temperature zone of a muffle furnace and heated to the target temperatures at a programmed rate of 10 °C/min. During degradation, specimens at 250 °C were removed and weighed every 24 h, while those at 300 °C and 350 °C were weighed every 3 h, so as to monitor mass loss dynamically. After degradation, the panels were transferred to a constant-temperature chamber and kept at room temperature for 24 h. Six degradation scenarios were designed in this study, designated according to the nomenclature XX-TYY-ZZ: XX denotes the resin matrix type (EP and SME), YY denotes the degradation temperature (T250, T300, T350), and ZZ denotes the degradation duration (3D for 3 days and 3H for 3 h). In addition, to establish the baseline of pristine mechanical properties, two non-degraded reference scenarios were included, designated as EP-T25 and SME-T25 for the two resin matrices, respectively (T25 denotes room temperature, 25 °C, with no thermo-oxidation). All scenario parameters are summarized in Table 2.

During the thermo-oxidative tests, the internal temperature of the panels was monitored in real time using K-type thermocouples coupled with a temperature acquisition system. The recorded data were compiled into temperature–time curves of the thermo-oxidative process, which were used to verify the consistency between the actual thermal history inside the furnace and the programmed profile, ensuring that the entire degradation process remained controllable and reproducible.

Scanning electron microscopy (SEM) was employed to characterize the cross-sectional micro-morphology of the post-degradation CFRP laminates. The sampling location was at the center region of the specimen to ensure the representativeness of the observations. Specimen preparation involved sequential polishing with sandpapers of various grit sizes, followed by gold sputtering on the observation surface.

### 2.4. Tensile and Compressive Mechanical Property Testing and Characterization

**Figure 3:**
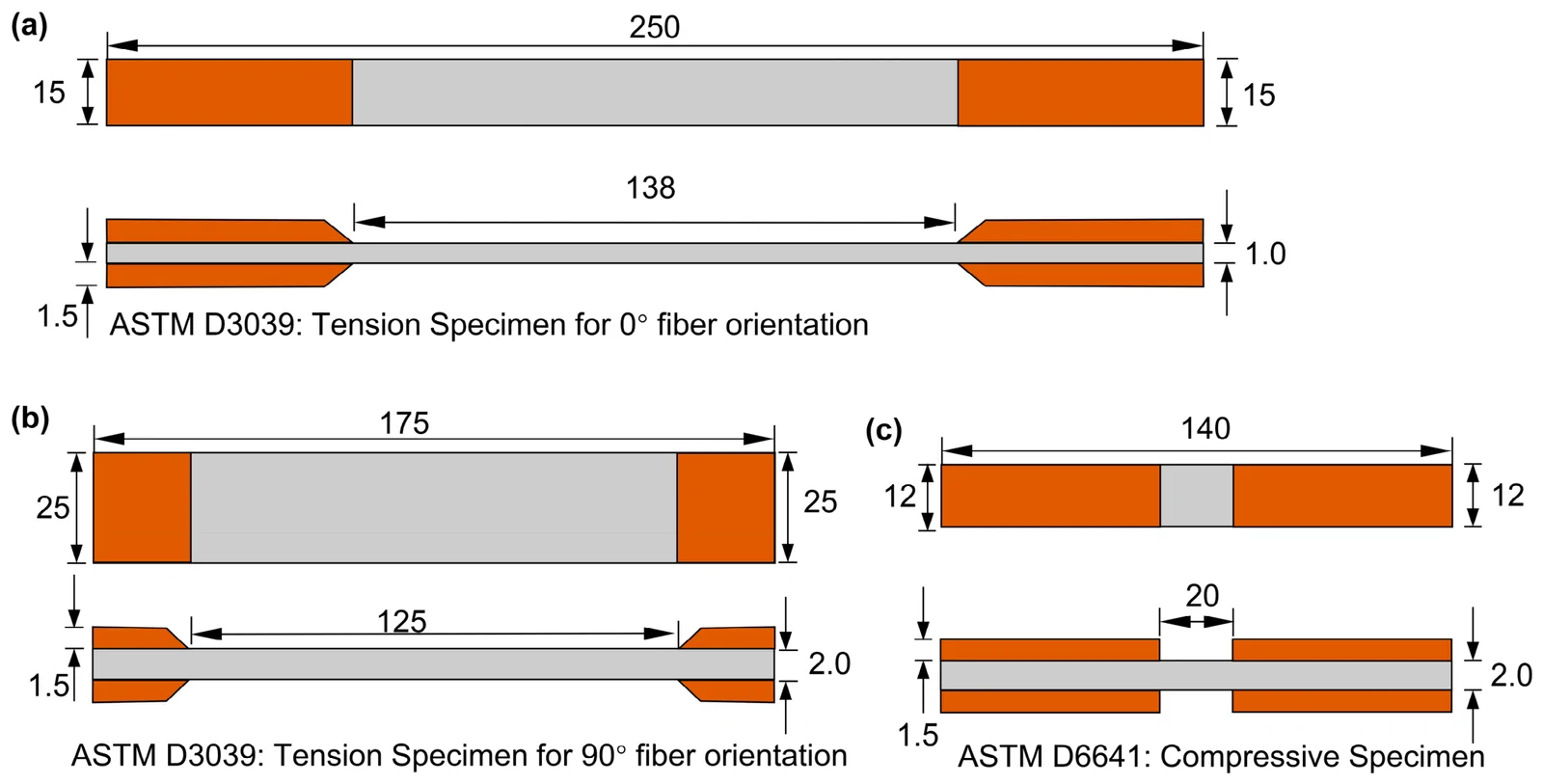
Geometric dimensions of the specimens for EP- and SME-based composites: (**a**) 0° tensile (ASTM D3039); (**b**) 90° tensile (ASTM D3039); (**c**) compressive (ASTM D6641).

The tensile and compressive mechanical properties of the composites were tested in accordance with ASTM D3039 [31] and ASTM D6641 [30] standards, respectively, and the corresponding specimen geometries are illustrated in Figure 3. All laminates were fabricated with identical processing parameters and showed statistically equivalent void contents (Section 2.2); the two laminate systems are therefore directly comparable.

**Figure 4:**
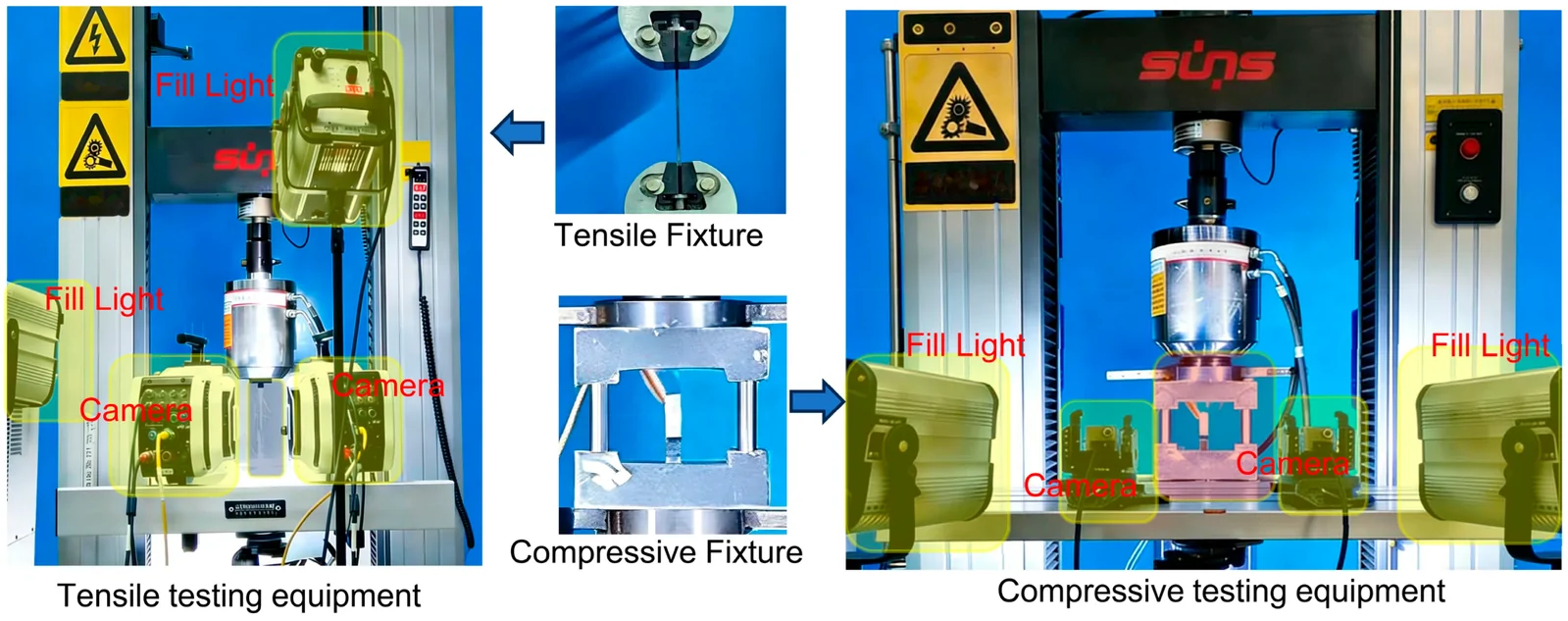
Tensile and compression testing apparatus and fixtures.

The tests were performed on a universal testing machine under displacement-controlled loading, with loading rates of 2 mm/min and 1 mm/min for the tensile and compressive tests, respectively. The axial strain during loading was measured by digital image correlation (DIC) rather than crosshead displacement, so that machine compliance errors are excluded. Prior to testing, a random speckle pattern was sprayed onto the gauge section of each specimen. A 3D DIC system (Vic-3D LS, Correlated Solutions, Inc., Irmo, SC, USA) was employed; although the system is capable of stereo measurement, only its 2D (single-camera) functionality was used here, since only the in-plane axial strain was required. Images were acquired by using a 12-megapixel Basler camera (model 12M, resolution 4096 × 3000) equipped with a 50 mm Schneider lens at a frame rate of 2 fps. The speckle images were analyzed using Vic-3D LS, a built-in post-processing module supplied with the testing equipment, and the axial strain was obtained from a virtual extensometer with a gauge length of 5 mm placed within the gauge section. The testing apparatus and fixture configurations are shown in Figure 4.

The elastic modulus was calculated from the load recorded by the testing machine and the DIC-measured axial strain. For each specimen, the stress–strain curve was constructed, and the modulus was determined by linear regression of the initial linear portion within a strain range of about 0.10–0.40%, in accordance with ASTM D3039/D6641 [30,31]. This interval corresponds to the linear elastic response of the material, prior to the onset of damage or nonlinear deformation. All specimen groups were processed using identical fitting intervals and data processing procedures, ensuring that the modulus comparison among different thermo-oxidative exposure conditions is based on a consistent methodology.

## 3. Results and Discussion

### 3.1. Comparative TGA/DTG Analysis of the Resins Before and After Modification

**Figure 5:**
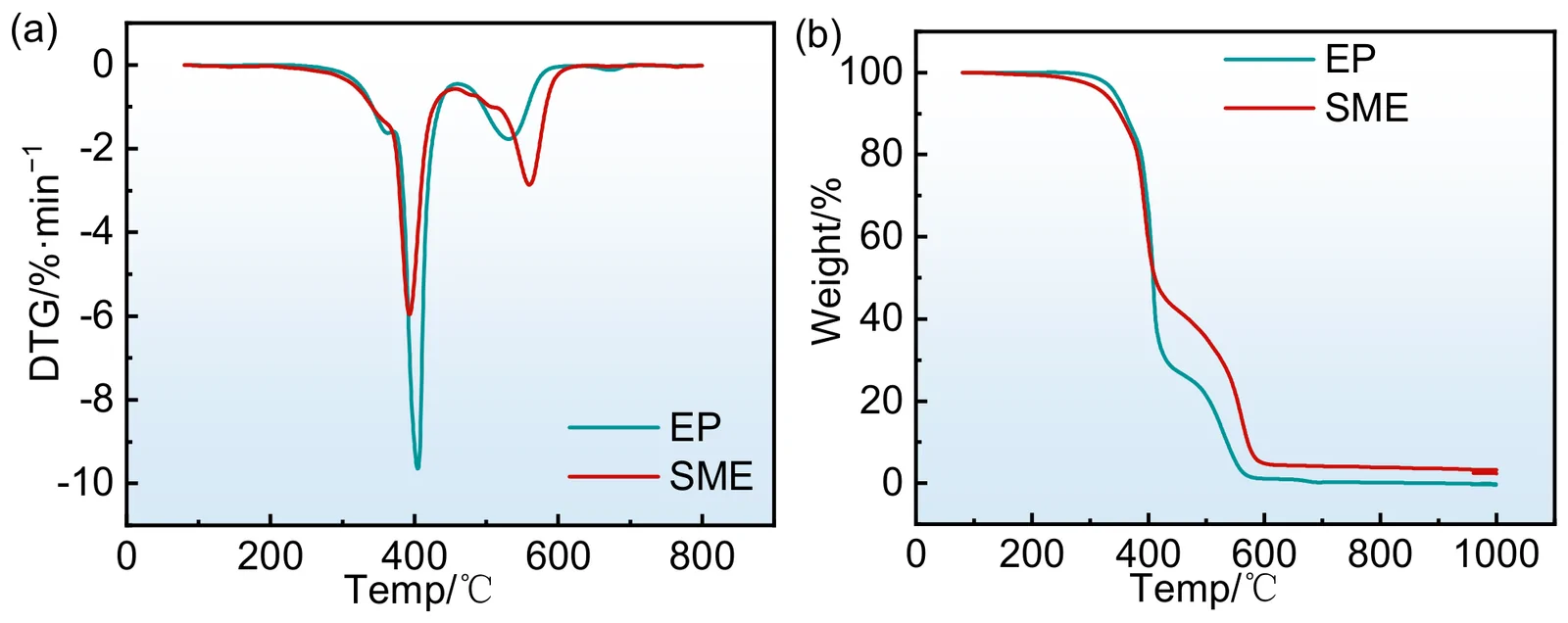
Thermogravimetric curves of neat and modified resins: (**a**) DTG curves; (**b**) TG curves.

Based on the TG–DTG curve analysis (see Figure 5), the thermo-oxidative degradation behavior of EP and SME in air can be divided into two main stages according to the temperature distribution characteristics.

Stage I (150–450 °C): main degradation (thermal decomposition) stage. This stage corresponds to the severe scission of the epoxy molecular backbone (including aromatic rings and crosslinked structures). EP exhibits a sharp and deep DTG peak at approximately 400 °C (about −10%·min^−1^), indicating the rapid rupture of macromolecular chains within this narrow temperature interval, accompanied by the concentrated release of small-molecule oxidation products and hence drastic mass loss. In contrast, the main thermo-oxidative peak of SME occurs in a similar temperature range but is substantially broadened with a markedly reduced peak height (about −6%·min^−1^), the peak rate being about 60% of that of EP. This demonstrates that silicone modification effectively suppresses the intensity of thermal cracking of the resin matrix. In the range of 250–350 °C, the DTG curve of SME shows an earlier and more pronounced increase in mass loss rate than that of EP, which may be attributed to the thermal rearrangement or early volatilization of some low-molecular-weight segments in the siloxane component, rather than large-scale scission of the epoxy backbone.

Stage II (450–650 °C): residual char oxidation stage. Both materials exhibit a secondary DTG peak near 580 °C, corresponding to the secondary oxidative decomposition in air of the char formed in Stage I. The minor difference between the two curves in this stage indicates that silicone modification has a limited influence on the high-temperature oxidation behavior of the final char.

Mechanism analysis and conclusions: During the main degradation stage, the high thermal stability of Si–O–Si bonds in SME and the dense crosslinked network formed thereby effectively hinder the thermal cracking and oxidation of C–O–C and C–C bonds in the epoxy backbone [33], resulting in a substantially reduced mass loss rate and a much milder thermo-oxidative process. It should be noted that the formation of this C–Si–O hybrid network has been confirmed in our previous work through FTIR (the emergence of Si–O–Si and Si–O–C bands and the systematic change of the C–OH band), ^1^H/^29^Si NMR (the appearance of Si–CH_2_ and CH_2_–O–Si signals and the disappearance of Si–OH), and epoxy value titration [21,26,28]; the TGA results therefore reflect the degradation response of an already-established network rather than inferring its formation from thermal analysis alone. Although SME exhibits slight early mass loss at lower temperatures, the main thermo-oxidative stage is the decisive factor governing the high-temperature service performance of epoxy resins. Judging comprehensively from the peak rate, peak width and degradation temperature interval of the DTG curves, silicone modification significantly improves the resistance to thermo-oxidative degradation at elevated temperatures.

### 3.2. Comparison of High-Temperature Thermo-Oxidative Degradation Behavior of CFRP Before and After Silicone Modification

**Figure 6:**
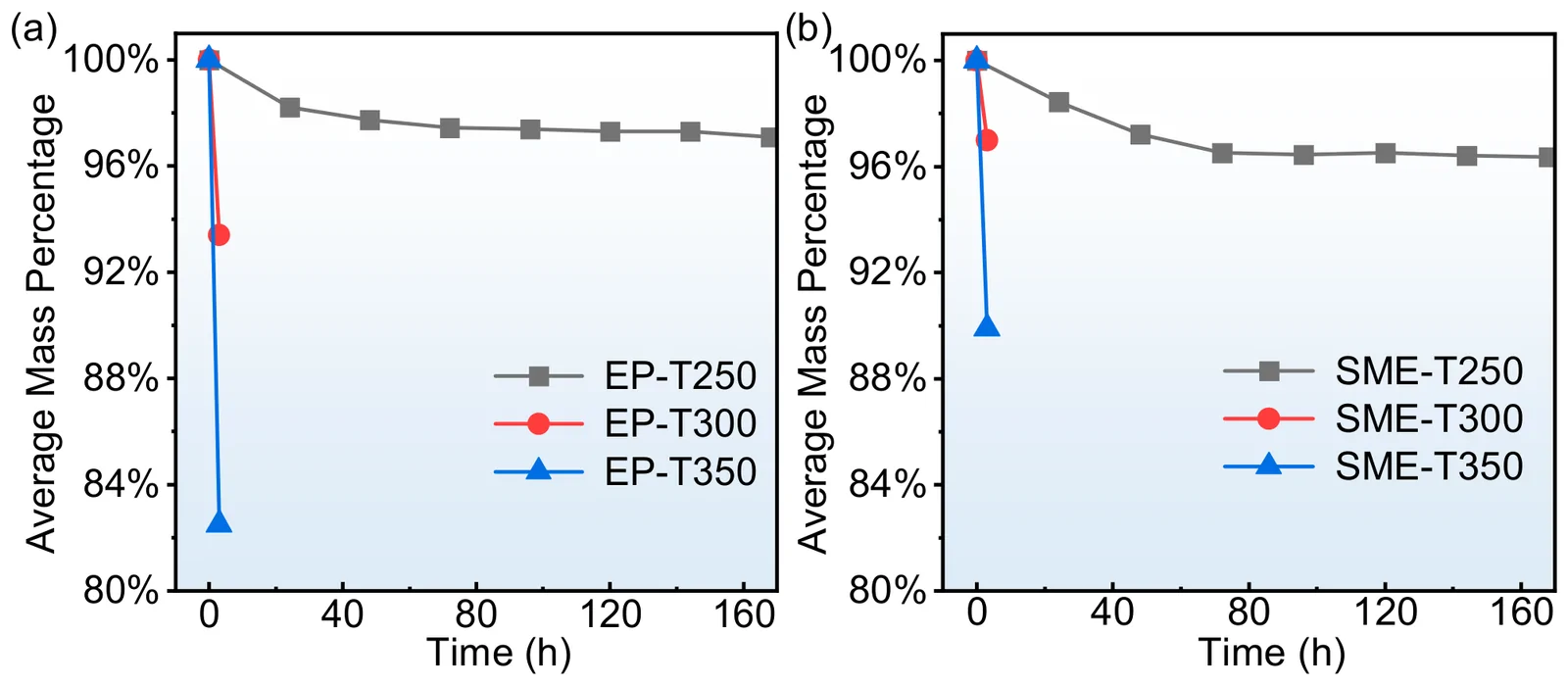
Mass loss ratio–time curves of 90° tensile CFRP laminates under thermo-oxidative degradation: (**a**) EP-based; (**b**) SME-based.

Figure 6 presents the evolution of mass loss ratio with exposure time under thermo-oxidative degradation for the two CFRP laminates at 250, 300 and 350 °C. Taking the 90° tensile specimens as an example, under the T250 condition, the mass loss ratios of the EP-based and SME-based composites after 24 h of the thermo-oxidative process were 1.98% and 2.45%, respectively, rising to 2.55% and 2.65% after 72 h, and 2.90% and approximately 2.65% at 168 h, respectively. Under the T300 condition, the mass loss ratios after 3 h of the thermo-oxidative process were 6.70% and 5.00%, respectively; under the T350 condition, the mass loss ratios after 3 h of the thermo-oxidative process were 17.50% and 10.10%, respectively.

The results indicate the following: (1) the degree of thermo-oxidative degradation, rather than exposure temperature or duration per se, governs the degradation behavior: as the mass loss increases from below 3% (T250-3D) to 5–7% (T300-3H) and 10–18% (T350-3H), the degradation state transitions from mild to severe. (2) Silicone modification significantly improves the high-temperature thermo-oxidation resistance of the composites. Under the conditions of 300 °C and 350 °C, the mass loss ratios of the SME-based composites are markedly lower than those of the EP-based counterparts, with the advantage becoming more pronounced at higher temperatures. At 350 °C, the mass loss ratio of the SME-based composite (10.10%) is reduced by approximately 42.3% compared with that of the EP-based composite (17.50%), demonstrating that the incorporation of silicone components effectively suppresses the high-temperature degradation of the resin matrix [34]. (3) Under prolonged exposure at the low temperature of 250 °C, the degradation rates of both materials decay substantially with increasing exposure time, and the mass loss tends to stabilize. The increment in mass loss ratio of the EP-based composite between 72 h and 168 h becomes markedly smaller, while that of the SME-based composite remains essentially constant after 72 h, indicating that low-temperature degradation is concentrated in the initial stage, after which the process is dominated by the slow release of residual volatiles and the overall thermo-oxidation reaction gradually approaches stagnation. The slightly higher early mass loss of SME at 250 °C is consistent with the early volatilization of low-molecular-weight siloxane segments revealed in Section 3.1, and does not compromise its advantage at higher temperatures. The marginal mass loss increment between 72 h and 168 h at 250 °C confirms that this regime is close to kinetic saturation; the observed property changes can therefore be attributed to the degradation degree rather than to further accumulation of exposure time. It is clarified that this observation applies only to the temperature range and exposure regimes of the present study and cannot be extrapolated as a universal threshold.

### 3.3. Post-Degradation Tensile and Compressive Behavior of Silicone-Modified and Neat Epoxy CFRP Laminates

This section focuses on evaluating the influence of thermo-oxidative degradation extent on the mechanical anisotropy of the materials, specifically covering the longitudinal (parallel to the fibers) and transverse (in plane, perpendicular to the fibers) tensile/compressive strengths and elastic moduli. Throughout this section, the percentages reported for the SME–EP comparison denote the relative increase in the SME value over the EP value under the same exposure condition.

#### 3.3.1. Longitudinal Tensile Properties and Failure Mode Analysis

**Figure 7:**
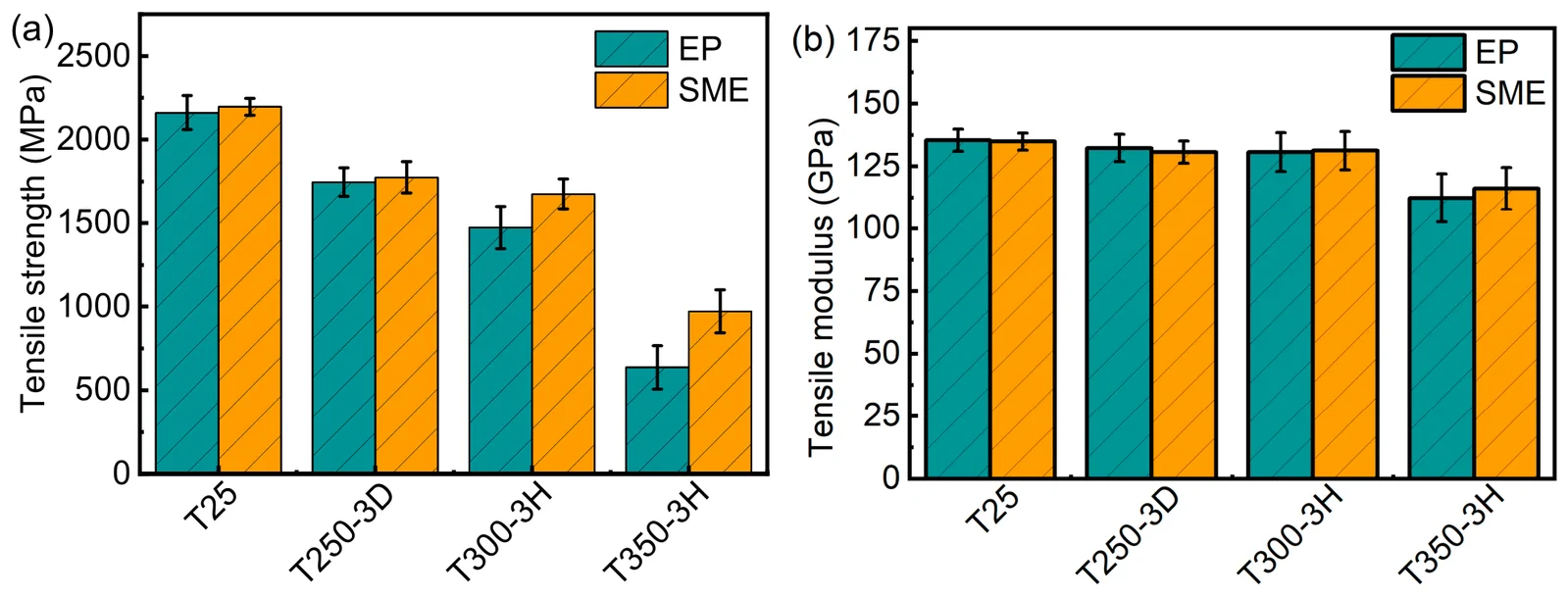
Longitudinal tensile properties (strength and modulus) of CFRP laminates based on neat epoxy (EP) and silicone-modified epoxy (SME): (**a**) EP-based; (**b**) SME-based.

Figure 7 presents the quantitative statistical results of the longitudinal tensile strength and elastic modulus of the CFRP laminates before and after silicone modification following thermo-oxidative treatment. The experimental results show that thermo-oxidative degradation extent exerts significant influences on the longitudinal tensile strength. With increasing thermo-oxidative temperature and duration, the longitudinal tensile strength exhibits a pronounced monotonic decreasing trend. In contrast, the effect of thermo-oxidative damage on the initial longitudinal tensile modulus is relatively limited, with only a slight reduction observed when the temperature reaches 350 °C; this retention of stiffness, however, is specific to the fiber-dominated longitudinal direction (see Section 3.3.2 and Section 3.3.3 for the compressive and transverse moduli). This phenomenon can be explained as follows: under longitudinal tensile loading, the carbon fibers serve as the primary load-bearing phase, and thermo-oxidative environments below 350 °C are insufficient to induce oxidation of the carbon fibers or significant stiffness degradation [35]. When the temperature reaches 350 °C, the lateral constraint effect of the resin matrix on the fibers is substantially weakened, and the resulting fiber lateral deflection and micro-slip contribute to a certain extent to the modulus reduction.

In addition, the silicone-modified resin markedly suppresses thermo-oxidative degradation-induced strength loss. Under the T250-3D, T300-3H and T350-3H scenarios, the longitudinal tensile strength of the modified-resin CFRP is improved by 1.6%, 13.6% and 52.7%, respectively, relative to the unmodified counterpart. These results indicate that silicone-modified resin can effectively retard the deterioration of longitudinal tensile properties under high-temperature thermo-oxidative degradation.

#### 3.3.2. Longitudinal Compressive Properties and Failure Mode Analysis

**Figure 8:**
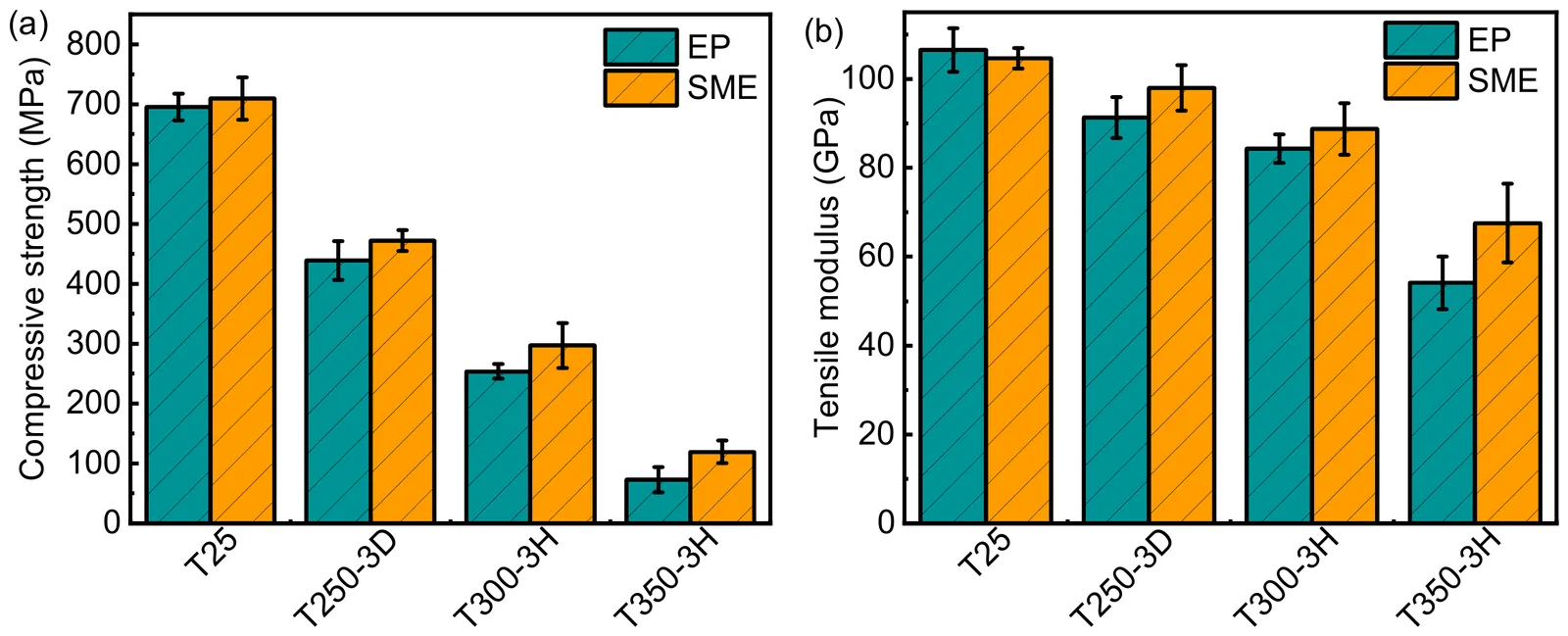
Longitudinal compressive properties (strength and modulus) of CFRP laminates based on neat epoxy (EP) and silicone-modified epoxy (SME): (**a**) EP-based; (**b**) SME-based.

Figure 8 presents the quantitative statistical results of the longitudinal compressive strength and elastic modulus of the CFRP laminates before and after silicone modification following thermo-oxidative treatment. The experimental results show that, compared with the longitudinal tensile strength, the influence of thermo-oxidative temperature and duration on the longitudinal compressive strength is more pronounced. With increasing thermo-oxidative temperature and duration, the longitudinal compressive strength exhibits a significant monotonic decay trend; compared with the longitudinal tensile modulus, the weakening effect of thermo-oxidative damage on the longitudinal compressive modulus is also more obvious. This phenomenon can be explained as follows: longitudinal compressive failure is more sensitive to the shear performance and modulus of the matrix; the thermo-oxidative interlaminar delamination and degradation of the matrix shear modulus trigger local buckling, which in turn leads to two macroscopic failure modes, namely splitting and kinking.

Furthermore, the silicone-modified resin plays a significant role in suppressing thermo-oxidative strength degradation. Under the T250-3D scenario, the longitudinal compressive strength of the SME-based CFRP is 6.9% lower than that of the EP-based CFRP; meanwhile, under the T300-3H and T350-3H scenarios, the longitudinal compressive strengths are improved by 17.1% and 63.2%, respectively, relative to the EP-based counterpart. These results indicate that, in high-temperature thermo-oxidative environments, silicone-modified resin can effectively retard the deterioration of longitudinal compressive properties.

#### 3.3.3. Transverse Tensile Properties and Failure Mode Analysis

**Figure 9:**
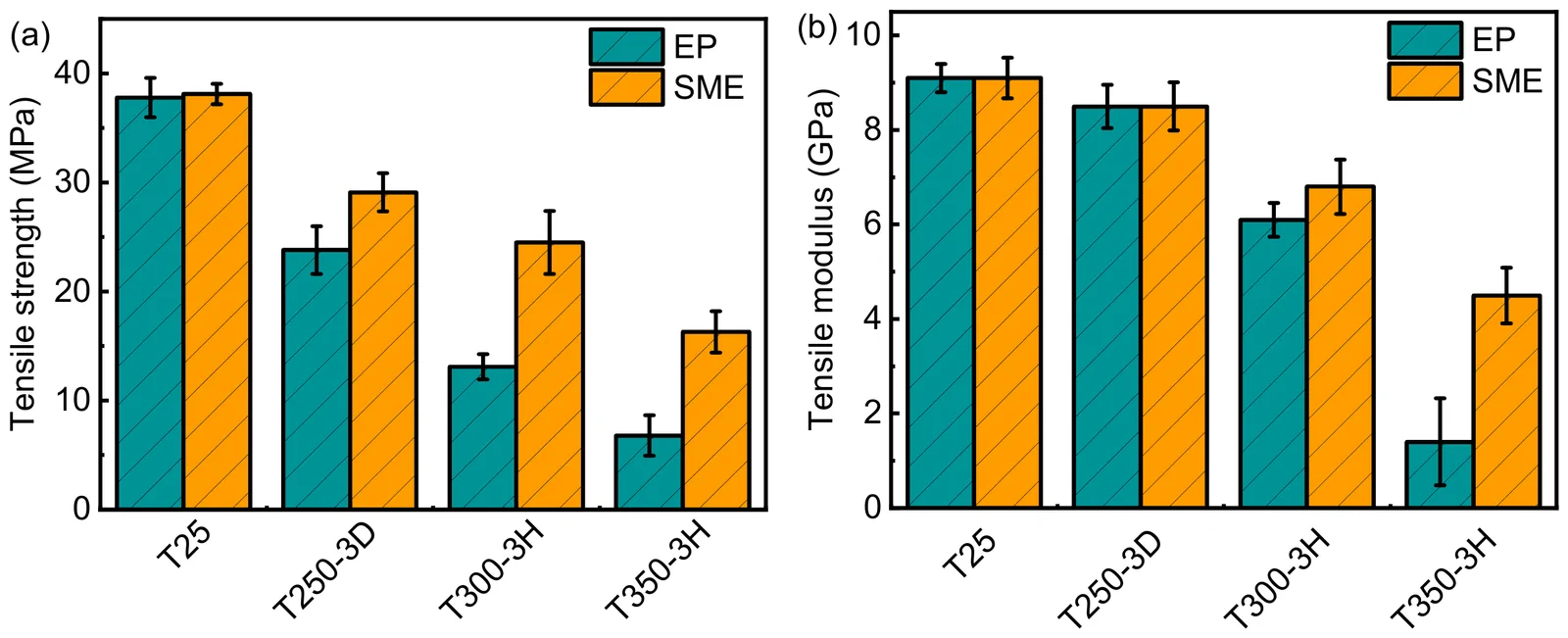
Transverse tensile properties (strength and modulus) of CFRP laminates based on neat epoxy (EP) and silicone-modified epoxy (SME): (**a**) EP-based; (**b**) SME-based.

Figure 9 quantitatively compares the transverse tensile properties of the EP-based and SME-based CFRP laminates before and after thermo-oxidative treatment. The results show that the thermo-oxidative degradation extent significantly influences the transverse tensile strength: with increasing thermo-oxidative temperature and prolonged holding time, the transverse tensile strength exhibits an overall monotonic decay trend. It is noteworthy that, compared with the longitudinal tensile modulus, thermo-oxidative damage exerts a more pronounced weakening effect on the transverse tensile modulus. The primary reason lies in the fact that transverse tensile loading is mainly borne by the matrix and the fiber–matrix interface; thermo-oxidative matrix carbonization and embrittlement, pore and microcrack initiation and propagation, and interfacial debonding significantly impair transverse stress transfer and the synergistic load-bearing capacity [36]. In contrast, longitudinal tensile properties are largely dominated by the continuous carbon fibers, whose high axial stiffness renders the longitudinal modulus relatively insensitive to matrix/interfacial thermo-oxidative damage.

A quantitative comparison of the two laminate systems further highlights the benefit of silicone modification. Even under the mild T250-3D condition, the SME-based CFRP attains a transverse tensile strength of 29.1 MPa, already exceeding the EP-based value (23.8 MPa) by 22.3%. Such a severity-dependent advantage indicates that the protective role of the siloxane network becomes increasingly prominent as matrix degradation intensifies. As the severity of thermo-oxidative degradation increases, this gap widens considerably: after 3 h at 300 °C and 350 °C, the SME-based laminates outperform their EP-based counterparts by 87.1% and 139.7%, respectively. Consequently, the higher the degree of thermo-oxidation, the more pronounced the relative increase in transverse tensile strength of the SME-based CFRP over the EP counterpart.

#### 3.3.4. Transverse Compressive Properties and Failure Mode Analysis

**Figure 10:**
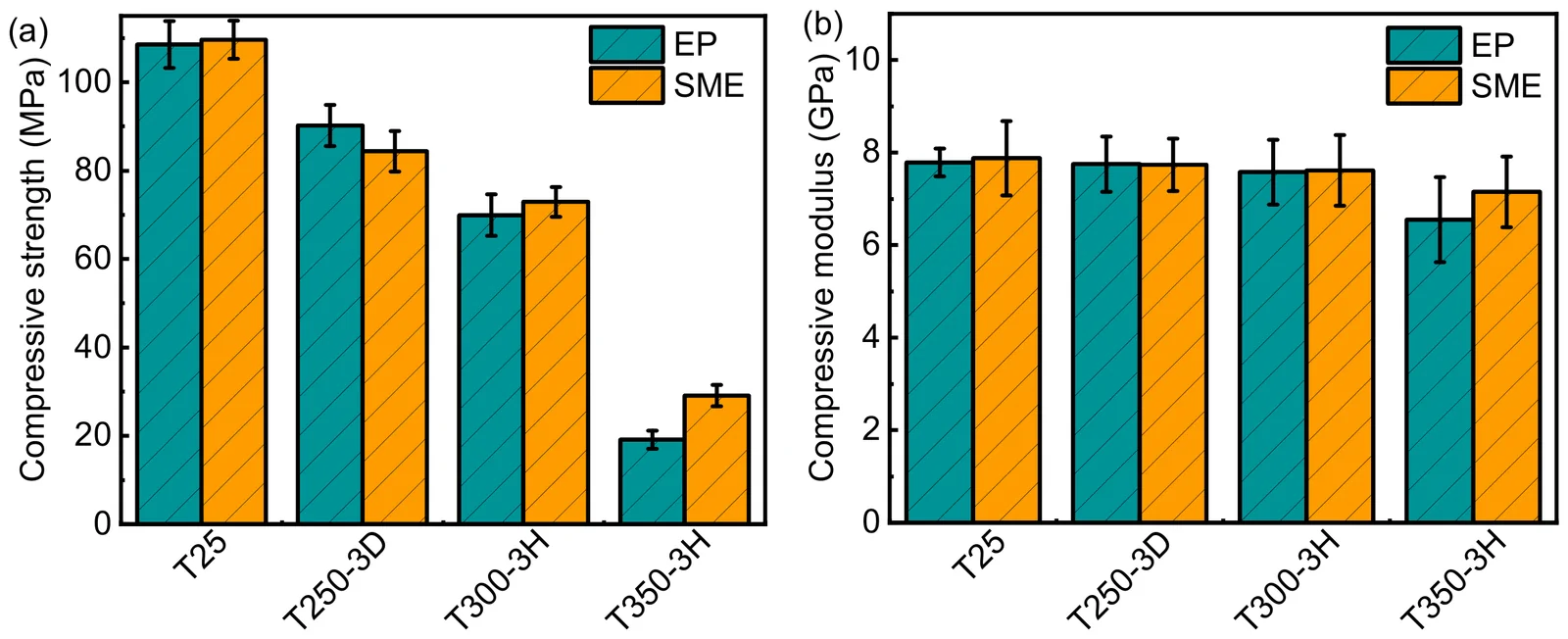
Transverse compressive properties (strength and modulus) of CFRP laminates based on neat epoxy (EP) and silicone-modified epoxy (SME): (**a**) EP-based; (**b**) SME-based.

Figure 10 quantitatively compares the transverse compressive properties of the EP-based and SME-based CFRP laminates before and after thermo-oxidative treatment. The results show that, within the investigated range, both increasing thermo-oxidative temperature and prolonged holding time aggravate the degradation of transverse compressive properties, with the transverse compressive strength exhibiting an overall monotonic decay trend. Specifically, the thermo-oxidative temperature primarily promotes matrix thermo-oxidation, carbonization and interfacial damage, while the holding time further governs the propagation of pores, microcracks and interfacial debonding; the two factors jointly determine the accumulated damage state. Compared with the transverse compressive strength, the transverse compressive modulus responds less sensitively to thermo-oxidative damage, indicating that from the initial to the moderate damage stage, the fiber skeleton and residual matrix can still maintain a considerable stiffness reserve, whereas strength degradation relies more heavily on the increase in defect density, the decline in interfacial stress transfer efficiency, and the weakening of local load-bearing and anti-buckling capacities. By comparison, the degradation of the neat epoxy-based (EP-based) specimens is more pronounced; owing to the introduction of the siloxane network, the SME matrix possesses higher thermal stability, toughness and interfacial fracture resistance, exhibiting higher strength and modulus retention rates and a gentler degradation slope under identical thermo-oxidative conditions, although this does not alter the general trend of monotonic property degradation with increasing degradation severity. It can therefore be concluded that the transverse compressive strength can serve as a sensitive indicator for characterizing the degree of thermo-oxidative damage, while the transverse compressive modulus is more suitable for reflecting the residual stiffness retention level; silicone modification can alleviate, to a certain extent, the thermo-oxidative degradation of transverse compressive properties.

### 3.4. Mechanistic Interpretation of the Performance Enhancement of Silicone-Modified CFRP Composites

**Figure 11:**
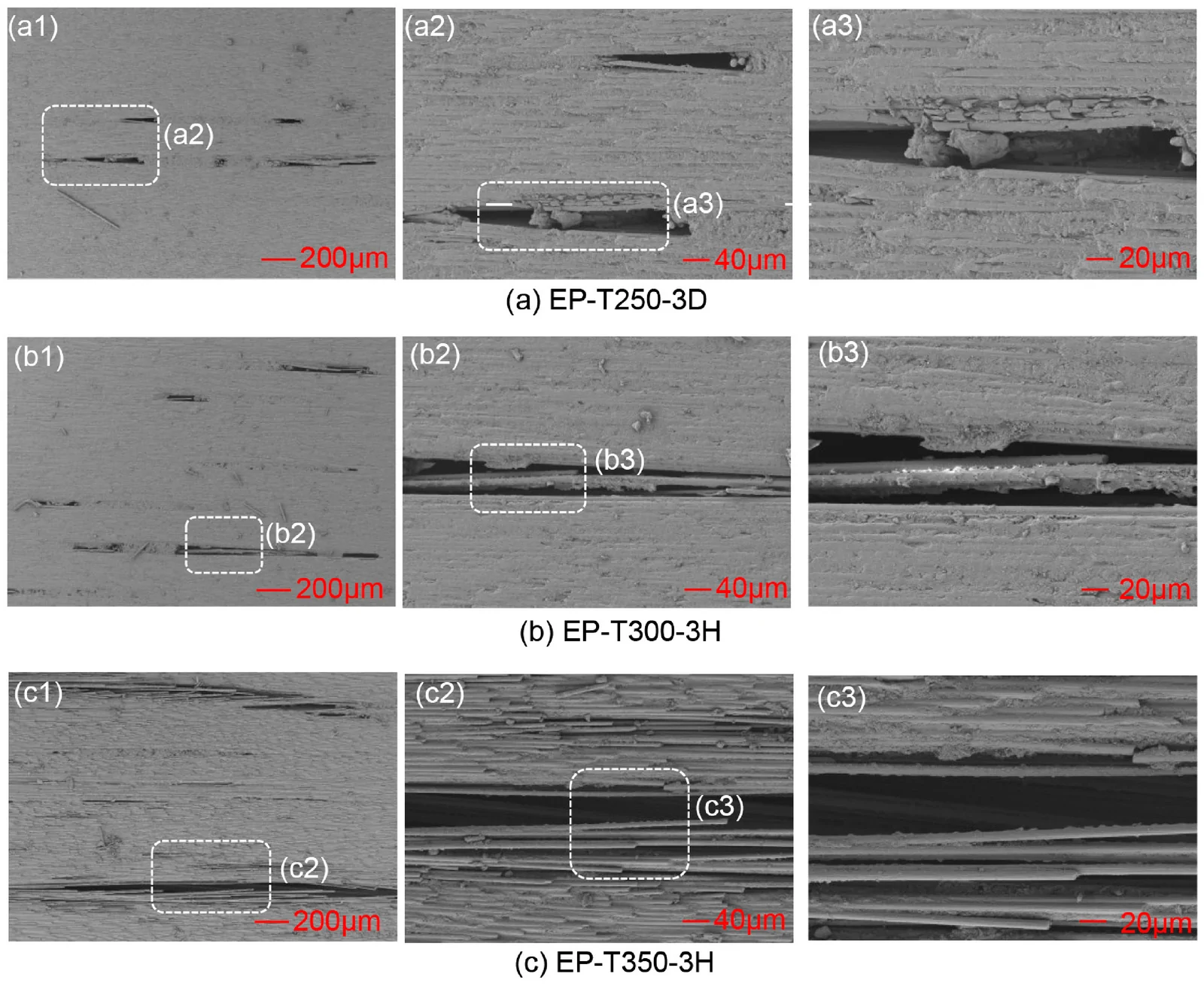
Microstructures and locally magnified views of EP-based composites after exposure at three temperatures: (**a**) 250 °C; (**b**) 300 °C; (**c**) 350 °C. (**a2**), (**b2**) and (**c2**) are 5× magnified views of the local regions marked in (**a1**), (**b1**) and (**c1**), respectively; (**a3**), (**b3**) and (**c3**) are 2× magnified views of (**a2**), (**b2**) and (**c2**), respectively.

**Figure 12:**
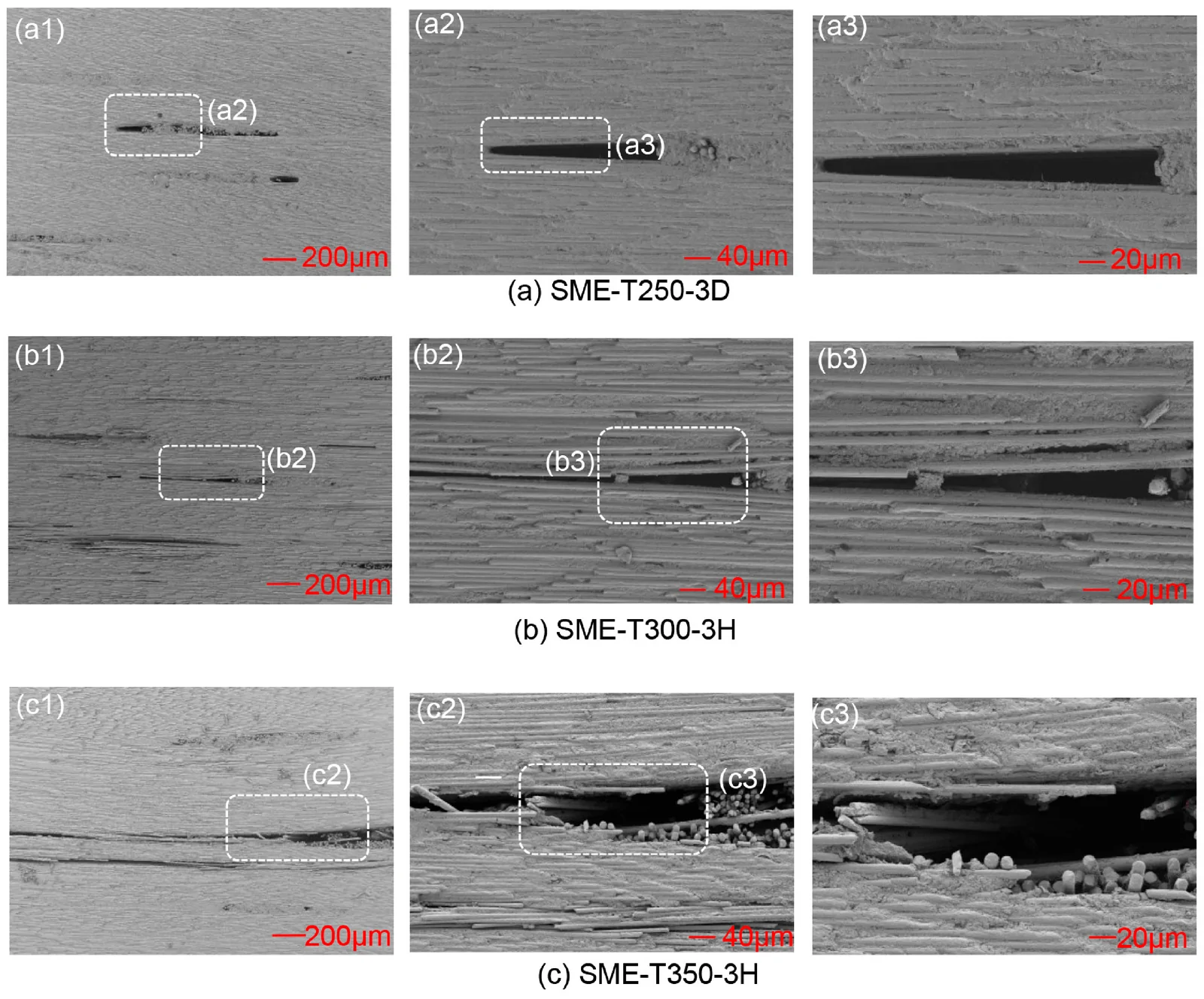
Microstructures and locally magnified views of SME-based composites after exposure at three temperatures: (**a**) 250 °C; (**b**) 300 °C; (**c**) 350 °C. (**a2**), (**b2**) and (**c2**) are 5× magnified views of the local regions marked in (**a1**), (**b1**) and (**c1**), respectively; (**a3**), (**b3**) and (**c3**) are 2× magnified views of (**a2**), (**b2**) and (**c2**), respectively.

Figure 11 and Figure 12 present the cross-sectional micro-morphologies of the unmodified and silicone-modified epoxy composites at different thermo-oxidative temperatures observed by scanning electron microscopy (SEM), focusing on the evolution of the fiber–matrix interface and the initiation and propagation of cracks, so as to reveal the differences in the microscopic damage-resistance mechanisms between the two systems. It is worth emphasizing that SEM provides morphological evidence (cracking, matrix degradation and debonding), whereas the protective role of the C–Si–O network invoked below is attributed to the chemically established structure in our previous work [21,26,28].

At the thermo-oxidative stage of 250 °C, initial microcracks propagating along the fiber–matrix interface appear in both materials, and the fiber surfaces are partially exposed over large areas, with interfacial debonding already being quite significant, indicating that matrix degradation and interfacial weakening intensify simultaneously. At a thermo-oxidative temperature of 300 °C, the damage discrepancy between the two systems widens markedly: the unmodified system exhibits a dramatic increase in crack density and propagation depth, a high degree of fiber–matrix separation, and extensively exposed fibers; meanwhile, the modified system is dominated by superficial microcracks, with the fiber–matrix interfacial bonding remaining in good condition and damage propagation being evidently restrained, reflecting the stabilizing effect of the C–Si–O hybrid network on the interface. At the thermo-oxidative stage of 350 °C, the damage in both systems reaches its peak: in the unmodified system, the fibers are detached from the matrix over large areas with no resin residue on the surfaces, and the interfacial functionality is essentially lost; in contrast, although cracks and local debonding also occur in the modified system, no obviously exposed fibers are observed, the interface remains intact in some regions, and the amount of residual resin is substantially higher, demonstrating a strong capacity for interfacial retention at elevated temperatures.

In summary, the silicone modification strategy effectively suppresses matrix degradation and interfacial debonding at elevated temperatures by virtue of the highly stable C–Si–O hybrid network, whose formation has been chemically confirmed in previous work [21,26,28], significantly delaying crack initiation and propagation. 

## 4. Conclusions

The post-degradation mechanical behavior and damage evolution mechanisms of CFRP laminates with silicone-modified epoxy (SME) and neat epoxy (EP) matrices were systematically investigated. The main conclusions are as follows:

(1) Silicone modification markedly improves the thermo-oxidation resistance of the epoxy matrix. TGA/DTG analysis reveals two thermo-oxidation stages—main decomposition (150–450 °C) and char oxidation (450–650 °C)—for both resins. The main degradation peak of SME is substantially broadened, with the peak mass 3wloss rate reduced from approximately 10%·min^−1^ (EP) to 6%·min^−1^, owing to the high thermal stability of Si–O–Si bonds and the dense crosslinked network that hinders the thermal scission of C–O–C and C–C bonds in the epoxy backbone.

(2) Mass loss during thermo-oxidative degradation is governed by the exposure temperature–time history through the resulting degradation degree, and silicone modification effectively suppresses degradation at elevated temperatures. Pronounced acceleration of the thermo-oxidation rate is observed at 300 °C compared with 250 °C under the investigated conditions. At 350 °C for 3 h, the mass loss of SME-based CFRP (10.10%) is approximately 42.3% lower than that of EP-based CFRP (17.50%). Under prolonged exposure at 250 °C, the mass loss rate of both systems decays substantially and stabilizes, indicating that low-temperature thermo-oxidation concentrates in the initial stage.

(3) Thermo-oxidative damage causes monotonic degradation of the anisotropic mechanical properties, which is effectively mitigated by silicone modification. The strengths in all four loading configurations (0°/90° tension and compression) decrease monotonically with increasing degradation severity, with transverse properties being more sensitive to thermo-oxidative damage than longitudinal ones. Under the severe T350-3H condition, the longitudinal tensile strength, longitudinal compressive strength and transverse tensile strength of SME-based laminates exceed those of the EP-based laminates by 52.7%, 63.2% and 139.7%, respectively. The degradation sequence of strength and stiffness is direction-dependent: under longitudinal tension, the strength degrades markedly whereas the modulus is relatively well retained, exhibiting a strength-before-stiffness sequence; under transverse tension and longitudinal compression, by contrast, the stiffness degradation is equally significant—with the transverse modulus dropping from approximately 9 GPa (T25) to about 1–2 GPa (T350-3H)—indicating that the transverse stiffness is most sensitive to matrix thermo-oxidation and interfacial damage.

(4) The strengthening mechanism originates from the dual suppression of matrix degradation and interfacial damage by the C–Si–O hybrid network. Multi-scale SEM observations show that increasing thermo-oxidation temperature induces matrix carbonization and embrittlement, microcrack initiation, and progressive fiber–matrix debonding. While EP-based laminates exhibit extensively exposed fibers and almost complete loss of interfacial functionality at 350 °C, SME-based laminates show markedly restrained crack propagation, no obvious fiber exposure, and substantially higher resin residue. The C–Si–O hybrid network preserves interfacial integrity, thereby delaying crack initiation and propagation and enhancing the thermo-oxidative damage tolerance of the composites.

In summary, silicone modification is an effective strategy for enhancing the high-temperature damage tolerance of epoxy-based composites, providing experimental evidence and data support for damage tolerance design, life prediction and safety assessment of composite structures in extreme thermal environments. It should be noted that strict decoupling of temperature and exposure time would require systematic isothermal time series at each temperature, which is recognized as an important extension of the present work. Future work will focus on establishing a quantitative correlation between thermo-oxidative mass loss and strength reduction coefficients, as well as multi-scale failure modeling under coupled thermo-mechanical loading.

## Figures and Tables

**Table 1:** Nomenclature and composition of resins and modified resins resins.

	EP/g	PPPS/g	4-MHHPA/g	DMP-30/g
EP	100	0	85.78	0.5
SME	80	20	47.09	0.35

Note: The curing agent and accelerator contents for SME were scaled according to the epoxy group content (80 g EP).

**Table 2:** A summary of thermo-oxidative degradation scenarios graded by degradation severity (characterized by cumulative mass loss in Section 3.2) and the corresponding test configurations for evaluating the post-degradation mechanical properties of CFRP laminates.

Test Group	Thermo-Oxidative Temperature (°C)	Thermo-Oxidative Time (h)	Number of Specimens
EP-T25	25	0	5
EP-T250-3D	250	72	5
EP-T300-3H	300	3	5
EP-T350-3H	350	3	5
SME-T25	25	0	5
SME-T250-3D	250	72	5
SME-T300-3H	300	3	5
SME-T350-3H	350	3	5

## Data Availability

The data supporting the reported results in this study are available within this article. Further inquiries can be directed to the corresponding authors.
